# Acetaminophen Attenuates Doxorubicin-Induced Cardiac Fibrosis Via Osteopontin and GATA4 Regulation: Reduction of Oxidant Levels

**DOI:** 10.1002/jcp.24367

**Published:** 2013-03-22

**Authors:** Kathryn J Schunke, Luke Coyle, Gary F Merrill, David T Denhardt

**Affiliations:** Department of Cell Biology and Neuroscience, Rutgers UniversityPiscataway, NJ

## Abstract

It is well documented in animal and human studies that therapy with the anti-cancer drug doxorubicin (DOX) induces fibrosis, cardiac dysfunction, and cell death. The most widely accepted mechanism of cardiac injury is through production of reactive oxygen species (ROS), which cause mitochondrial damage, sarcomere structural alterations, and altered gene expression in myocytes and fibroblasts. Here we investigated the effects of acetaminophen (APAP, *N*-acetyl-*para*-aminophenol) on DOX-induced cardiac injury and fibrosis in the presence or absence of osteopontin (OPN). H9c2 rat heart-derived embryonic myoblasts were exposed to increasing concentrations of DOX ± APAP; cell viability, oxidative stress, and OPN transcript levels were analyzed. We found a dose-dependent decrease in cell viability and a corresponding increase in intracellular oxidants at the tested concentrations of DOX. These effects were attenuated in the presence of APAP. RT-PCR analysis revealed a small increase in OPN transcript levels in response to DOX, which was suppressed by APAP. When male 10–12-week-old mice (OPN^+/+^ or OPN^−/−^) were given weekly injections of DOX ± APAP for 4 weeks there was substantial cardiac fibrosis in OPN^+/+^ and, to a lesser extent, in OPN^−/−^ mice. In both groups, APAP decreased fibrosis to near baseline levels. Activity of the pro-survival GATA4 transcription factor was diminished by DOX in both mouse genotypes, but retained baseline activity in the presence of APAP. These effects were mediated, in part, by the ability of APAP, acting as an anti-inflammatory agent, to decrease intracellular ROS levels, consequently diminishing the injury-induced increase in OPN levels.

J. Cell. Physiol. 228: 2006–2014, 2013. © 2013 Wiley Periodicals, Inc.

Doxorubicin (DOX), an anthracycline antibiotic also known as Adriamycin, has been widely used in the treatment of a variety of cancers. However, its anti-neoplastic application is limited by its side effects, which result in cardiac dysfunction and fibrosis (Chen et al., 2007; Suliman et al., 2007; Simunek et al., 2009; McTiernan, 2011). The most widely accepted mechanism of cardiac injury is via reactive oxygen species (ROS), which induce mitochondrial damage, DNA strand breaks, sarcomere structural alterations, and altered gene expression.

Approximately 26% of patients develop heart failure after cumulative DOX treatment (Swain et al., 2003). The manner in which the left ventricle heals and remodels after injury is a major determinant of eventual cardiac function and the progression to heart failure. Cardiac adaptation in response to intrinsic or extrinsic stress involves a complex process of chamber remodeling, including changes in cardiac gene expression leading to structural changes in the myocardial wall. Physiologically, ventricular remodeling is a compensatory response that enables the heart to adapt to increased stress, but can quickly turn maladaptive and pathologic if the balance of collagen synthesis and degradation is not tightly regulated.

ROS may contribute to the remodeling process in a number of ways, including inducing synthesis of cytokines such as osteopontin (OPN), which participates in reconfiguration of the extracellular matrix, and by contributing to myocyte loss via apoptosis or other cell death mechanisms (Denhardt et al., 2001; Zohar et al., 2004). OPN is a secreted phosphoprotein found in body fluids (e.g., plasma, urine, milk) and the matrix of mineralized tissues. Its expression is minimal in normal adult cardiomyocytes and fibroblasts, but increases during pathological events such as tissue remodeling after injury (Klingel and Kandolf, 2010; Singh et al., 2010). It is known to foster cell survival and tissue regeneration in the injured heart (Turakhia et al., 2007; Mori et al., 2008; Hunter et al., 2012). Waller et al. (2010) have reviewed the role of OPN in cardiovascular disease, emphasizing its potential as a therapeutic target.

ROS activate several protein kinase pathways that likely mediate the response of the heart to DOX exposure. Mitogen activated protein kinases (MAPKs) have been proposed as cellular mediators linking DOX to the apoptotic pathway and the myocardial remodeling pathway (Chen et al., 2007; Suliman et al., 2007; Turakhia et al., 2007; Simunek et al., 2009; Singh et al., 2010; Thandavarayan et al., 2010). GATA4, a member of the zinc finger transcription factor family, important in regulating differentiation, sarcomere synthesis, and cell survival, is stimulated by ROS in part via MAPK-dependent activation (Zheng and Blobel, 2010).

Previous studies in the Merrill laboratory have shown that APAP has cardio-protective properties during oxidant challenge (Merrill and Goldberg, 2001; Merrill et al., 2001; Merrill, 2002; Hadzimichalis et al., 2007; Jaques-Robinson et al., 2008). The aim of the current study was to investigate the effects of APAP on the cellular physiology of the heart following DOX-induced oxidant challenge. We hypothesized that APAP would attenuate DOX-induced ROS levels and subsequent chamber fibrosis by reducing OPN synthesis and preserving GATA4 function.

## Materials and Methods

### Materials

Doxorubicin hydrochloride (2 mg/ml in sterile saline for injection, TEVA Parenteral Medicines Inc., Irvine, CA) was kindly provided by Dr. Michael Reiss (Cancer Institute of New Jersey). Acetaminophen was obtained from Fisher Scientific (Pittsburgh, PA). All other reagents were from Gibco (Grand Island, NY) unless indicated otherwise.

### Cell culture

H9c2 rat heart-derived embryonic myocytes were obtained from American Type Culture Collection (ATCC CRL-1446) and cultured in Dulbecco's modified essential medium (Gibco; Carlsbad, CA) supplemented with 10% fetal bovine serum, 2 mM glutamine, 100 units/ml penicillin, 100 µg/ml streptomycin, and 1 mM sodium pyruvate in humidified 5% CO_2_ in air at 37°. Cells were kept at or below 70% confluence during culture and allowed to reach 80–90% before experimentation.

### Animals

OPN^−/−^ mice in the 129 background were bred and maintained along with isogenic OPN^+/+^ controls in the Animal Care and Use Facilities at Rutgers University, which is accredited by the Association for Assessment and Accreditation of Laboratory Animal Care and overseen by board-certified veterinarians. Institutional review and approval of protocols was obtained before initiating experiments. Our laboratory complies with the Guide for the Care and Use of Laboratory Animals. Male 10–12-week-old 129 mice (OPN^+/+^ or OPN^−/−^) were randomly assigned into four groups: (1) saline, (2) APAP (30 mg/kg), (3) DOX (4 mg/kg), or (4) DOX plus APAP (all in saline). Intraperitoneal injections were made weekly for 4 weeks. Following the fifth week, all mice were euthanized by CO_2_. The hearts were then subjected to histological, immunohistochemical, and molecular biological analyses.

### H9c2 myoblasts

Cells were treated with PBS alone or supplemented with APAP (50 µM) for 15 min, incubated with DOX (2–8 µM in PBS) for 6 h, washed and then switched to fresh medium. At this point, cell viability was assessed, ROS were quantified, and OPN mRNA abundance was measured as described below.

### Cell viability assay

The viability of cultured cells was evaluated by performing a 3-(4,5-dimethylthiazol-2-yl)-2,5-diphenyltetrazolium bromide (MTT; Sigma; St. Louis, MO) assay as described (Turakhia et al., 2007). Briefly, after cells were treated with DOX ± APAP, MTT (5 mg/ml MTT in Hanks Buffered Salt Solution (HBSS; Gibco; Carlsbad, CA)) was added to a final concentration of 0.25 mg/ml and incubated with the cells for 4 h in the dark at 37°C. Cells with active mitochondria transform MTT into purple formazan crystals. The crystals were solubilized in 50 µl DMSO for 1 h. Absorbance of the samples was read using a microplate reader at 570 nm.

### Measurement of intracellular ROS production: 2′,7′-dichlorodihydrofluorescein

The determination of intracellular hydroperoxide production is based on the oxidation of 2′,7′-dichlorofluorescein diacetate (DCFH-DA; Sigma–Aldrich; St. Louis, MO) to the fluorescent 2′,7′-dichlorofluorescein (DCF). For quantification of cellular fluorescence, cells were incubated in phenol red-free DMEM in a 12-well plate containing 5 µM DCFH for 30 min. Cells were washed with HBSS and the medium replaced. Baseline measurements were taken for all wells using a microplate reader at an excitation of 485 nm and emission of 530 nm. Cells were then treated with DOX ± APAP, and measurements were taken every hour for 6 h. For visual images, cells were treated as above, but images were acquired after 2 h of treatment with DOX ± APAP using a fluorescence microscope with FITC filter.

### RT-PCR (reverse transcription-polymerase chain reaction)

Total RNA from H9c2 cells was isolated using the Trizol Reagent (Invitrogen; Carlsbad, CA). Briefly, 1 ml Trizol was added per 10-cm^2^ plate and allowed to sit for 5 min at room temperature. RNA was purified according to the manufacturer's instructions. The final preparation of RNA was tested for purity by spectrophotometry and accepted at 260/280 > 1.8. A 20-µl reaction volume consisting of a 2.5 mM (each) 2'-deoxynucleoside 5'-triphosphate mix (dNTP; Invitrogen), 5 µM N6 random hexamer (Integrated DNA Technologies; Coralville, IA), 5 µg of the RNA of interest in sterile H_2_O, was added to a 200-µl PCR microcentrifuge tube (Phenix Research Products; Hayward, CA) and heated at 65°C for 5 min in a thermal cycler. Then, 4 µl of 5× first strand buffer and 2 µl of 1 M dithiothreitol (Invitrogen) were added to the reaction, which was incubated at 25°C for 10 min. Next, 1 µl (200 U) Superscript II (Invitrogen) was added and incubated at 37°C for 50 min. The reaction was inactivated by heating to 70°C for 15 min. The negative control lacked Superscript II.

Four microlitres of complementary DNA (cDNA) were amplified in a reaction mixture containing 5 µl of 10× PCR buffer (200 mM Tris–HCl pH 8.4, 500 mM KCl), 1.5 µl of 50 mM MgCl_2_, 4 µl of 2.5 mM dNTP mix, 2 µl (10 units) of Taq DNA polymerase, 4 µl of amplification primers targeting the gene of interest, and 30.5 µl H_2_O for a final volume of 50 µl. The PCR products were analyzed by electrophoresis on a 1.5% agarose gel in Tris–acetate–EDTA buffer (TAE; 0.04 M Tris–acetate, 1 mM EDTA, pH 8.5). Densitometric analysis of ethidium-bromide-stained (0.5 µg/ml) agarose gels was performed using Image J software (NIH; Bethesda, MD). The ratio between the yield of each amplified product and that of the co-amplified internal control allowed a relative estimate of mRNA levels in the sample analyzed. The internal control was GAPDH, a housekeeping gene whose PCR product did not overlap with the OPN product. The two negative controls included a complete reaction setup lacking either cDNA template or Taq DNA polymerase. For OPN amplification we found optimal transcript measurements at 56.5° for 42 cycles using primers:

forward, 5′-ACGAGTTTCACAGCCATGAGGACA-3′;

reverse, 5′-GCAGTGGCCA TTTGCATTTCTTGC-3′

designed using IDT primerQuest software (Coralville, IA).

### Histological analysis

After the fifth week of DOX treatment, mice were euthanized; the hearts were removed and fixed in 10% buffered formalin overnight. Hearts were embedded in paraffin, after which 7-µm thick transverse sections were cut using a microtome and plated on poly-l-lysine slides (Sigma). The sections were then rehydrated in an ethanol series and stained with Sirius Red. Nuclei were stained for 1 min with Weigert's hematoxylin, washed, and then stained for 1 h in 0.1% Sirius Red stain (Direct Red 80, Sigma) in saturated picric acid. Slides were washed twice in 5% acetic acid, dehydrated in an ethanol series, and cleared with xylene. Sections were cover-slipped with Permount (Sigma). Images were collected using bright-field microscopy at 10–20 × magnification. Digital images (9–12 each) of the left ventricular free wall of sections from the apical third, middle, and basal third of each heart were assessed for fibrotic area using Image J software (NIH). The collagen area fraction (CAF) was measured by dividing the collagen area by the total tissue area of each image.

### Immunohistochemistry

The presence of phosphorylated (s105) GATA4 in the heart tissue was carried out using the streptavidin–biotin-peroxidase complex immunohistochemical technique. Slides were deparaffinized in xylenes and rehydrated in a graded ethanol series. The antigen retrieval was done by incubating slides in antigen retrieval buffer at 100°C (1:100; Vector Labs; Burlingame, CA) for 5 min at full power then 25 min at 10% power, followed by cooling at room temperature for another 20 min. After three washes in PBS, endogenous peroxidase activity was inactivated by incubating the slides for 30 min in 0.3% (v/v) H_2_O_2_ in the dark. Slides were then incubated in blocking buffer (PBS, 10% goat serum, 0.5% tween-20) for 1 h at room temperature. The slides were incubated overnight at 4°C with rabbit anti-GATA4 (1:200; phospho s105; Abcam; Cambridge, MA), washed 3× for 10 min in PBS and incubated for 1.5 h at room temperature with biotinylated anti-rabbit secondary antibody. They were then washed 3× with PBS and incubated with Avidin Biotin Complex (Vectastain; Vector Labs) for 30 min. Slides were washed with PBS, then diaminobenzidine (DAB; Vector Labs) was applied for 10 min as the chromogen. Slides were counterstained with hematoxylin for 30 sec and covered with a mounting solution (Vectamount; Vector Labs). In each run a negative control (where primary antibody was replaced with blocking buffer and only secondary antibody was used) were also included. Activated GATA4 was revealed by a brown color, as a result of the DAB reaction with the streptavidin-conjugated peroxidase solution. The assessment of the stained tissues was done in a blind fashion. For quantification, the number of DAB+ nuclei was divided by the total number of nuclei in the tissue section.

### Statistical analysis

All data are presented as mean ± standard error of the mean (SEM). The Student's *t*-test for non-paired replicates was used to identify statistically significant differences between treatment means. Group variability and interaction were compared using either one-way or two-way ANOVA followed by Bonferroni's post-tests to compare replicate means. Significance was accepted at *P* < 0.05.

## Results

### Cardiomyocyte viability

**Fig. 1:**
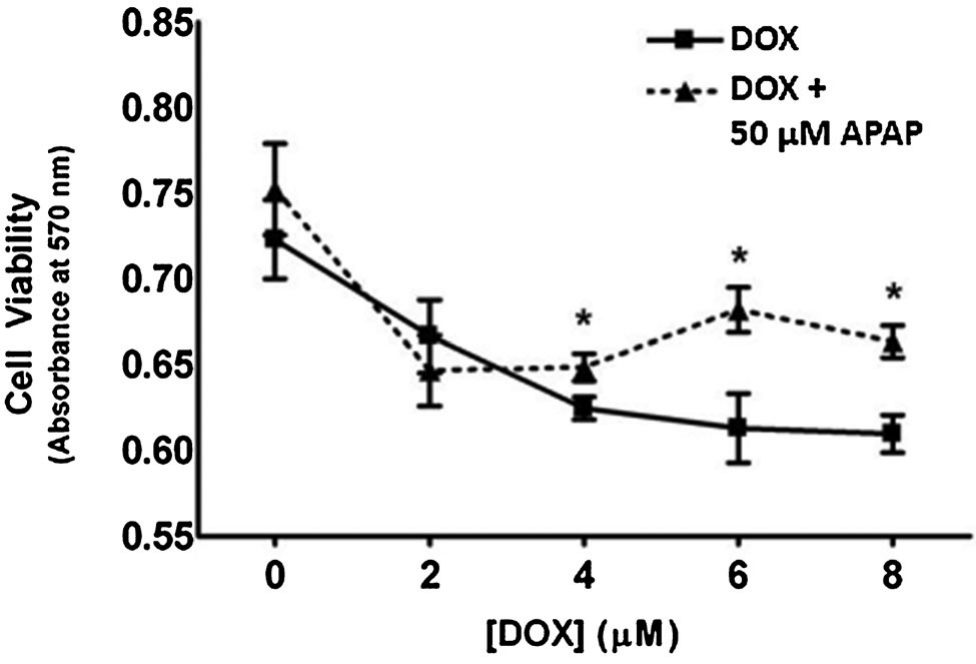
Effect of APAP (50 µM) on H9c2 cell viability after treatment with increasing concentrations of DOX, as determined by the MTT assay. Cells grown to 80% confluence were subject to DOX ± APAP for 6 h, the medium was changed, and the cells allowed to incubate for an additional 18 h. Cell viability was substantially (*P* < 0.001) decreased in DOX concentrations at and above 4 µM. APAP significantly (*P = 0.0065) preserved cell viability in DOX concentrations at 4 μM and above. Results represent means of six independent experiments ± SEM.

Doxorubicin toxicity was assessed using the MTT assay and H9c2 cells. Untreated control cells and cells treated with 50 µM APAP did not differ significantly from each other. A dose-dependent decrease in cell viability was observed in the tested range of DOX concentrations (Fig. 1). In the presence of DOX + APAP, cells receiving 6 µM (91% viability vs. 85% viability) and 8 µM (88% viability vs. 83% viability) DOX showed significantly increased viability in the presence of 50 µM APAP as compared to DOX treatment alone.

### Oxidative stress in Cardiomyocytes

**Fig. 2:**
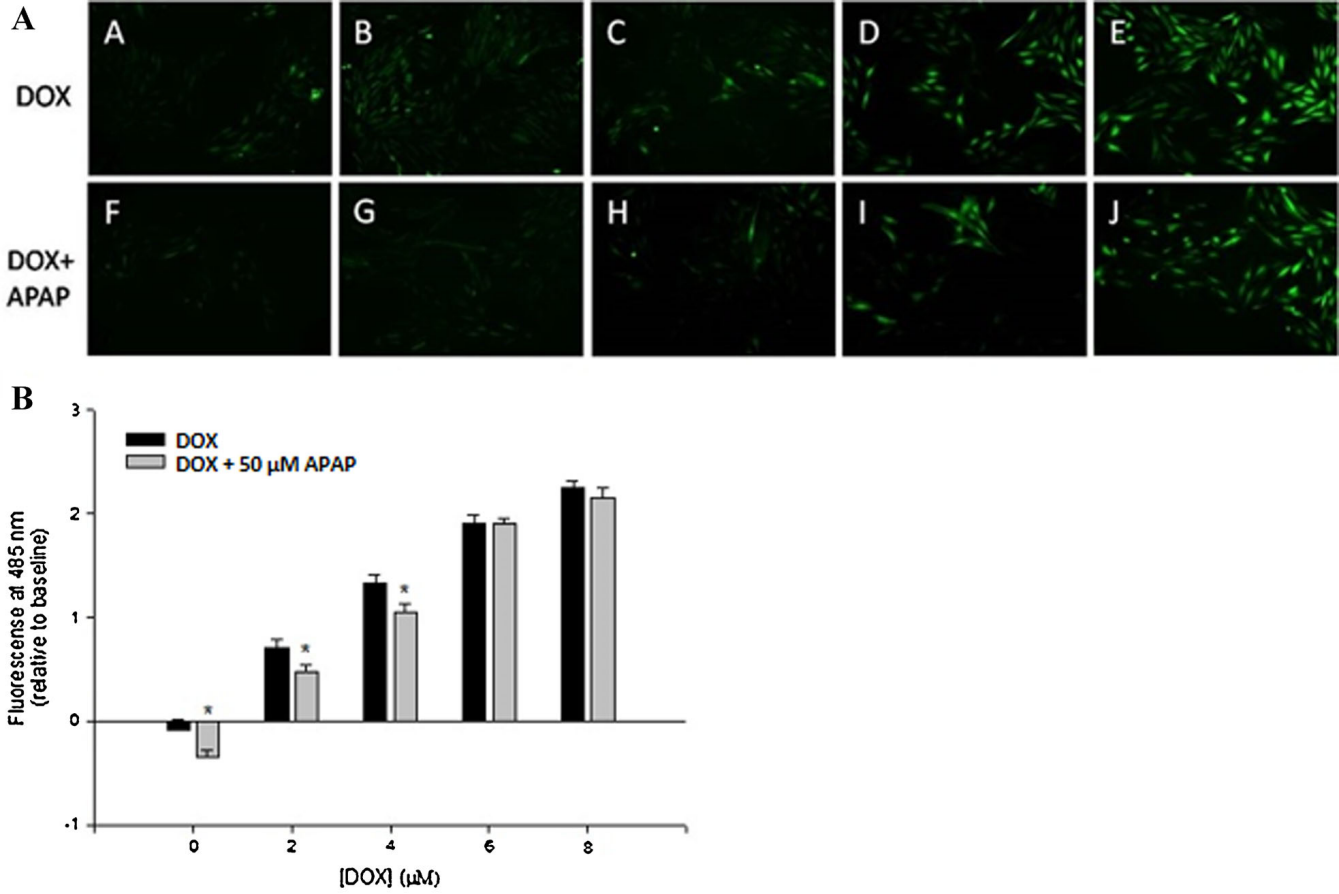
Increased intracellular oxidant levels in H9c2 cells exposed to DOX. Cells were incubated with increasing concentrations of DOX (A–E, F–J) and DOX+50 μM APAP (F–J): (A, F) 0 μM DOX, (B, G) 2 μM DOX, (C, H) 4 μM DOX, (D, I) 6 μM DOX, (E, J) 8 μM DOX for 2 h, then loaded with DCFH-DA and viewed under a fluorescence microscope. A: Fluorescent images positively correlate increased [DOX] to intracellular oxidation, an effect that was diminished in the presence of APAP. B: Cellular fluorescence was quantified using a plate reader. Results represent means of four independent experiments ± SEM. *P < 0.05.

Enhanced ROS levels in cells exposed to DOX were confirmed in H9c2 cells by quantifying intracellular oxidation of DCFH-DA. Cells were exposed to 2 µM, 4 µM, 6 µM, and 8 µM DOX for 6 h in the presence or absence of APAP (50 µM). A dose-dependent increase in DCFH-DA oxidation was evident in all cells after 1 h of treatment; this increase was reduced in the presence of APAP (Fig. 2A). A trend of increasing DOX-induced autophagic vacuoles was also observed (data not shown), which was similarly attenuated by APAP treatment. Quantification of mean DCF fluorescence intensity in control and treated cells was also performed. The results confirmed a significant increase in DCFH-DA oxidation at all concentrations of DOX, relative to baseline measures (Fig. 2B). At 2 µM and 4 µM concentrations of DOX, cells treated with DOX + APAP showed a significant decrease in oxidant damage as compared to DOX alone. The same trend is seen at 6 µM and 8 µM DOX, although these differences did not achieve statistical significance.

### OPN transcript levels

**Fig. 3:**
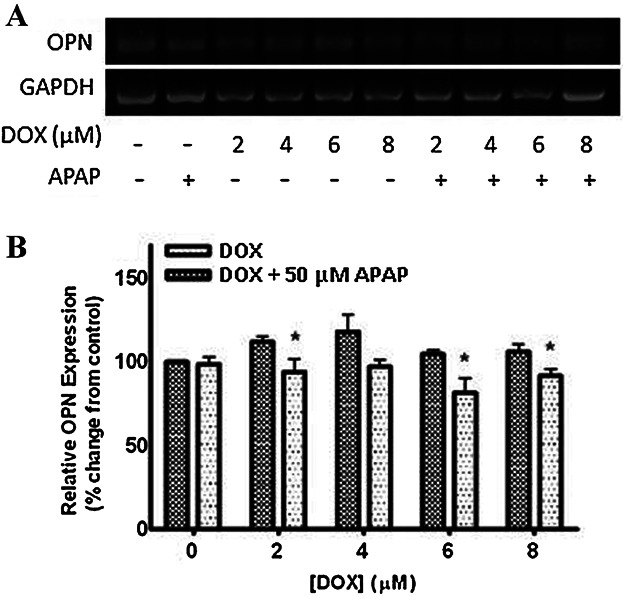
Osteopontin transcript levels in H9c2 cells increase in response to DOX, an effect that is attenuated in the presence of APAP. A: Effect of APAP on OPN mRNA levels after treatment with increasing concentrations of DOX evaluated by RT-PCR using rat gene-specific primers for OPN. Cells grown to 80% confluence were subject to DOX ± APAP for 6 h, medium was changed, and cells were allowed to incubate for an additional 18 h in fresh medium. B: Histogram shows DOX-induced OPN transcript abundance, which was significantly attenuated by APAP at all concentrations of DOX. Results represent means of four independent experiments ± SEM. *P < 0.05.

To determine whether regulation of OPN mRNA abundance is influenced by DOX, transcript levels were measured in H9c2 cells by RT-PCR. A small DOX-dependent increase in OPN transcript level was seen after treatment with 2 µM, 4 µM, 6 µM, and 8 µM DOX. Cells treated with DOX + APAP showed reduced OPN mRNA abundance relative to those treated with DOX alone (Fig. 3). When cells were treated with DOX alone, OPN mRNA levels increased maximally to 118% of baseline levels (4 h), but when treated with DOX in the presence of APAP, OPN mRNA decreased to 82% (6 h) of baseline.

### DOX-induced fibrosis

**Fig. 4:**
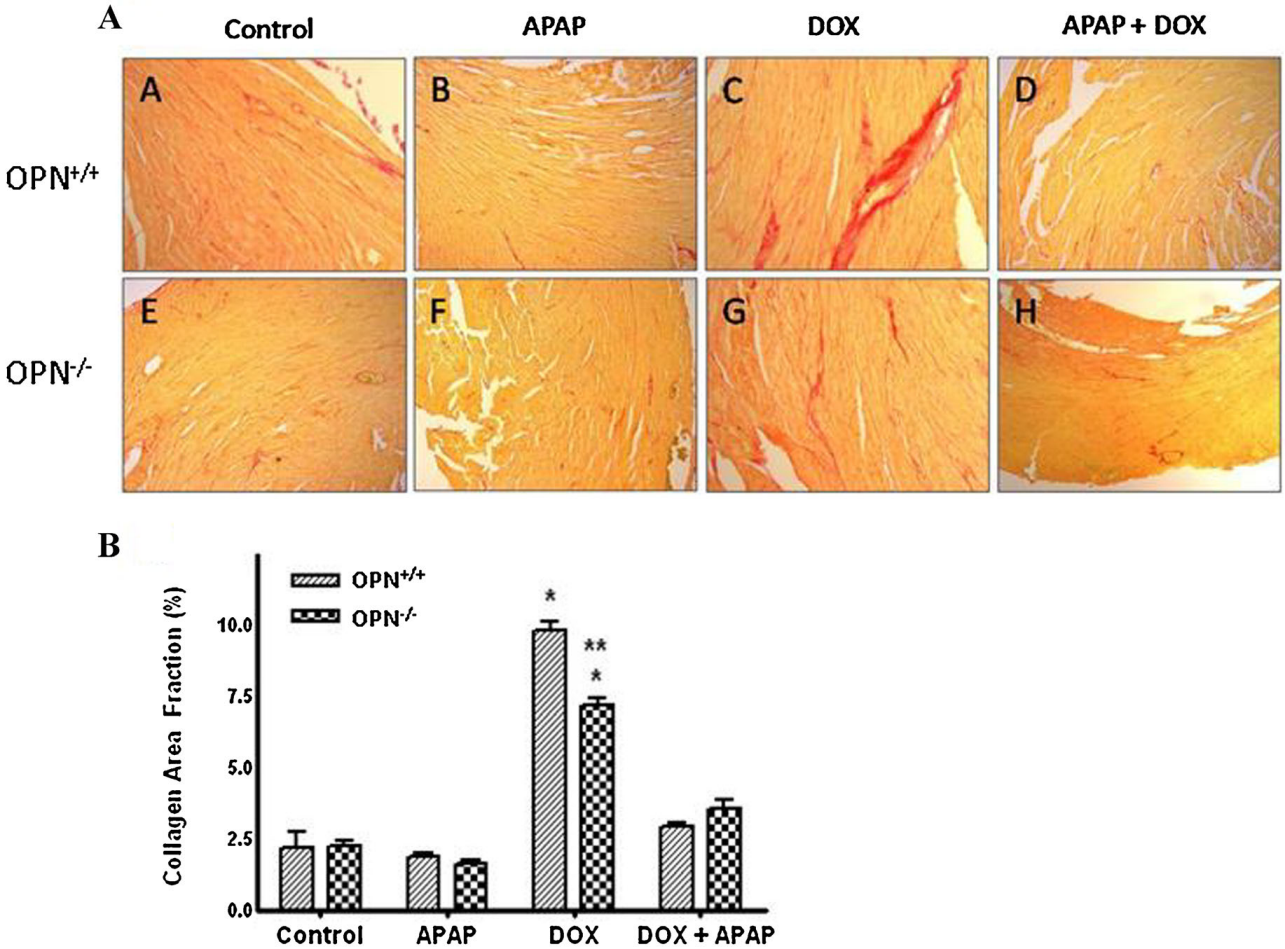
Left ventricular cardiac fibrosis in OPN^+/+^ and OPN^−/−^ mice. A: Fibrosis is evident in PFA-fixed paraffin embedded transverse tissue sections as revealed by Sirius red staining within the LV free wall of DOX-treated mice. OPN^−/−^ mice have significantly less left ventricular fibrosis after DOX treatment. Fibrosis in OPN^+/+^ and OPN^−/−^ mice is significantly attenuated by APAP. Sirius red staining: (A) OPN^+/+^ control, (B) OPN^+/+^ APAP, (C) OPN^+/+^ DOX, (D) OPN^+/+^ DOX + APAP, (E) OPN^−/−^ control, (F) OPN^−/−^ APAP, (G) OPN^−/−^ DOX, (H) OPN^−/−^ DOX + APAP. B: Quantification of collagen content (collagen area fraction). *P < 0.05 relative to control. **P < 0.05 relative to DOX treated OPN^+/+^.

Figure 4A illustrates the collagen content in the free wall of the left ventricle (LV) of the heart as indicated by the Sirius red-stained tissue portions. We assessed myocardial fibrosis using Sirius red-stained sections, and found no significant difference in collagen content between control hearts of OPN^+/+^ or OPN^−/−^ mice. Nor was there a difference between control and APAP (30 mg/kg) treated OPN^+/+^ or OPN^−/−^ mice. After the cumulative dose of 16 mg/kg DOX over 5 weeks, we found a fourfold increase in fibrosis in OPN^+/+^ mice, and a threefold increase in OPN^−/−^ mice compared to control groups (Fig. 4B). However, groups treated with APAP + DOX had significantly less collagen content than groups treated with DOX alone (OPN^+/+^: 2.9 ± 0.1% vs. 8.9 ± 0.3%; OPN^−/−^: 3.6 ± 0.3% vs. 7.8 ± 0.3%; *P* < 0.05). Additionally, fibrosis was significantly higher in the DOX group of OPN^+/+^ mice as opposed to OPN^−/−^ mice (8.9 ± 0.3% vs. 7.8 ± 0.3%; *P* < 0.05).

### GATA4 regulation

**Fig. 5:**
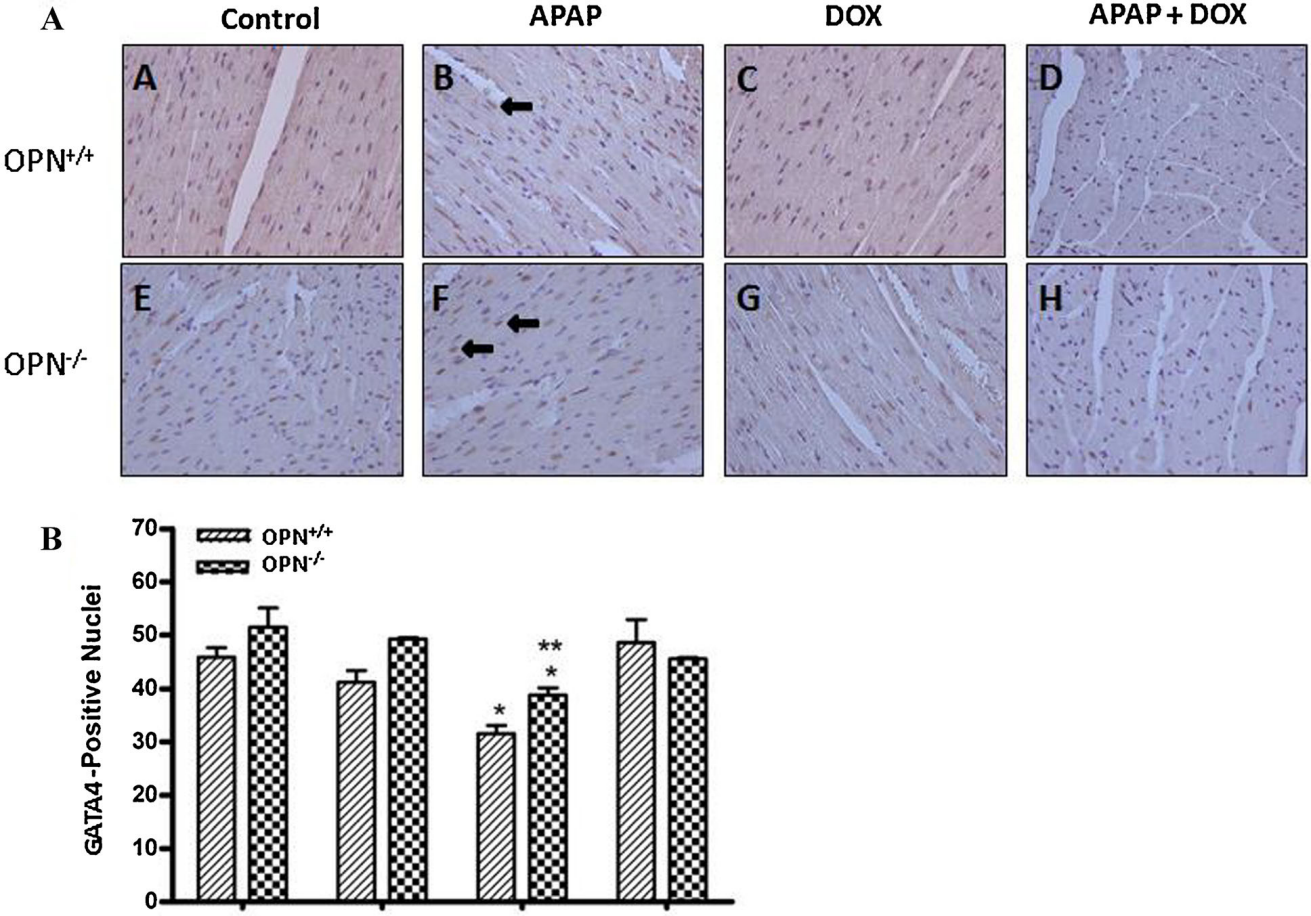
GATA4 expression in left ventricular tissue. A: Positive nuclear labeling with an antibody specific for serine 105 is seen in left ventricular free wall tissue in all treatment groups (A–H). Expression is significantly decreased in DOX treated mice (C, G), an effect which is attenuated in the presence of APAP (D, H). Moreover, OPN^−/−^ mice maintained significantly higher expression during DOX treatment, compared to OPN^+/+^ (G vs. C). Arrows indicate nuclei containing activated GATA4 (brown color) versus nuclei containing unactivated GATA4 that show only hematoxylin staining. B: Quantification of GATA4-positive nuclei. Average number of GATA4 positive nuclei per image area; 12 images per mouse. Groups: (1) saline, (2) APAP, (3) doxorubicin, (4) doxorubicin plus APAP. *P < 0.05 relative to control. **P < 0.05 relative to DOX-treated OPN^+/+^.

The number of DAB-positive nuclei (indicating GATA4 activation) in the left ventricle of OPN^+/+^ and OPN^−/−^ control mice were similar (46 ± 1.7 and 51.5 ± 3.6, respectively; Fig. 5). After DOX treatment, the number of DAB-positive nuclei fell to 67% of baseline in OPN^+/+^ mice, while OPN^−/−^ mice maintained 75% of baseline activation. Furthermore, in the presence of APAP and DOX, both OPN^+/+^ and OPN^−/−^ mice maintained a significantly higher fraction of activated GATA4 (106% and 88%, respectively) relative to the control.

## Discussion

### Experimental protocols

Experiments were conducted to find concentrations of DOX that would elicit oxidative stress in the cells and would approximate levels of DOX seen transiently in plasma following therapeutic use in patients (Green and Leeuwenburgh, 2002). The dose of 30 mg/kg APAP chosen for the mice in this study was based on the indicated therapeutic range for APAP in humans of 10–100 µg/ml plasma, which corresponds to a dose of about 1 g every 4 h for approximately four doses in a 50–70 kg patient (Rumack, 2004). The concentration of 50 µM used for H9c2 experiments was chosen based on previous literature utilizing a 10–500 µM range, and through pilot experiments investigating 10–200 µM APAP.

### Reactive oxygen species

In the present study, we used DOX to induce intracellular oxidant injury, which we measured using FITC fluorescence (Fig. 1). Our observation (Fig. 2) of decreased oxidant stress in the presence of APAP confirms reports in the literature using other models of ROS-inducing injury. For example, Van Dyke et al. (1998) found that acetaminophen is a potent inhibitor of peroxynitrite-mediated chemiluminescence, and Merrill (2002) found that coronary effluent samples from acetaminophen-treated hearts exposed to ischemia and reperfusion had reduced levels of peroxynitrite relative to controls. Using hypoxic/reoxygenated guinea pig hearts, Rork et al. (2004) demonstrated significantly decreased peroxynitrite-mediated chemiluminescence in coronary effluent samples, and decreased superoxide-mediated lucigenin chemiluminescence. These reports confirm the ability of acetaminophen to reduce oxidant levels in injured cardiac tissue. Whaley-Connell and Sowers (2012) have reviewed evidence that agents that attenuate oxidative stress, induced for example by proinflammatory/profibrotic ROS, improve cardiac function.

Acetaminophen (Tylenol) is widely used clinically. It has a long-standing history of safety in humans when taken as prescribed. It is universally available and very inexpensive compared to current therapy for heart disease. Moreover, the FDA has recently approved use of intravenous acetaminophen (≤15 mg/kg) when other analgesics are either ineffective or contraindicated. Also acetaminophen is currently being used in children and neonates undergoing cardiopulmonary bypass surgery (http://clinicaltrialsfeeds.org/clinical-trials/show/NCT01228305, Simpson SA, 2012).

### Cell viability & oxidative stress in doxorubicin-treated cells

Doxorubicin targets rapidly dividing tumor cells, specifically by inhibiting topoisomerase action and suppressing DNA and RNA synthesis via intercalation in the DNA helix. These anticipated causes of cell death are not directly experienced by heart cells, as myocytes are largely quiescent. In vivo, the major DOX-induced injury sustained by heart cells is the result of elevated ROS levels, superoxide in particular, generated by NOX2 NADPH oxidase likely mediated by activation of angiotensin II (Zhao et al., 2010). For in vitro studies, we chose H9c2 cells, which are cardiac myoblasts that proliferate rapidly, unlike their differentiated in vivo counterparts. Our results (Figs. 1 and 2) show that APAP significantly attenuated DOX-induced cell death, resulting from increased intracellular oxidative stress, at concentrations of 4–8 µM. We therefore conclude that the increased viability of APAP-treated cells is significantly attributable to the decreased extent of oxidant-induced cell death.

Interestingly, we see in Figure 2 an apparent preservation of cell viability at high concentrations of DOX yet no significant reduction in oxidative stress at these concentrations. We suggest that is because the number of viable cells in cultures treated only with DOX is significantly decreased when compared to DOX + APAP, leaving fewer viable cells to contribute to the measured fluorescence. This would account for the decrease in fluorescence when compared to the DOX + APAP treated cells, which have a significantly increased viability and therefore more cells to experience oxidative stress and add to the fluorescence.

### Ventricular remodeling: ROS and fibrosis

The pro-fibrotic effects of ROS, generated in significant part by members of the (NAD(P)H) oxidase family, are well recognized (Barnes and Gorin, 2011). They include increased fibroblast proliferation and transformation into myofibroblasts, expression of pro-fibrotic genes, and alterations in the balance between extracellular matrix destruction (mediated by matrix metalloproteinases) and formation (collagen synthesis). The ultra-structural changes seen in endomyocardial biopsies of patients with DOX-associated heart failure include loss of myofibrils, disarray of sarcomere structure, dilation of the sarcoplasmic reticulum, swelling of the mitochondria and cytoplasmic vacuolization (Unverferth et al., 1981). It is believed that DOX-induced cardiotoxicity involves the loss of cardiac myocytes in a dose-dependent manner. Given the limited regenerative capacity of the heart, when cumulative toxicity surpasses a threshold of reparable damage, a generic process of ventricular remodeling is triggered. Here we have documented for the first time, to our knowledge, the anti-fibrotic effects of APAP in the cardiovascular system (Fig. 4). Although the mechanism of action remains to be fully clarified, it appears that it is the suppression of NAD(P)H oxidase activity (Zhao et al., 2010) that mediates much of the protection since our results align with those using gene or drug therapeutics that reduce oxidant burden (Swain et al., 1997; Spallarossa et al., 2004, 2006). These observations illustrate the anti-inflammatory action of APAP. Similarly, Tripathy and Grammas (2009) reported an anti-oxidant and anti-inflammatory action of APAP, mediated by the induction of pro-survival Bcl2 expression, on neurons in culture subjected to the oxidant stressor menadione.

### Ventricular remodeling: OPN and fibrosis

Low levels of OPN are expressed in healthy cardiac muscle tissue. However, in inflammatory or pathological states, OPN expression in the heart increases markedly in a variety of cell types. Increased expression of OPN is known to be associated with the development of heart failure, both in primary pathological states such as pressure/volume overload, acute myocardial infarction, LV hypertrophy and various other cardiomyopathies (Graf et al., 1997; Trueblood et al., 2001). Conditional overexpression of OPN in mouse cardiomyocytes causes chronic myocarditis and premature death within 16 weeks (Renault et al., 2010). Although cardiac myocytes occupy 75% of normal heart tissue volume, they only account for 30–40% of the total cell number. The remainder of the cells consists mostly of fibroblasts, endothelial cells, immune cells and vascular smooth muscle cells, all of which can make OPN when appropriately stimulated.

In an induced myocardial infarct model using OPN^+/+^ and OPN^−/−^ mice, the expression of OPN in infarct regions was primarily localized to nonmuscle and infiltrating cells (Trueblood et al., 2001). Diffuse OPN message was also detectable in the non-infarct LV, with more focal message associated with blood vessels, presumably in endothelial and/or smooth muscle cells. In spontaneously hypertensive rats with heart failure, in situ hybridization of heart sections revealed abundant expression of OPN mRNA, primarily in non-myocytes in the interstitial and perivascular space (Singh et al., 1999). In hamsters with heritable cardiomyopathy, increased OPN expression was observed mainly in infiltrating macrophages and was localized to the interstitium (Murry et al., 1994). Immunohistochemical analysis of myocardial biopsies obtained from patients with heart failure due to dilated cardiomyopathy demonstrated increased OPN expression in cardiac myocytes, which correlated positively with impaired left ventricle (LV) function (Stawowy et al., 2002), and collagen type I levels (Satoh et al., 2005). More recently, Yu et al. (2009) demonstrated parallel increases in IL-18 levels, OPN expression, and interstitial fibrosis in murine models of left ventricular pressure and volume overload.

A well-organized extracellular matrix is essential to maintaining the strength and organization of the cardiac tissue. Okamoto and Imanaka-Yoshida (2012) and Frangogiannis (2012) have reviewed the importance of OPN, and other matricellular proteins, in repairing heart tissue after injury. Mice lacking OPN grow normally, but, after injury, often deposit collagen in an abnormal manner during wound healing (Liaw et al., 1998). Using scanning electron microscopy, Trueblood et al. (2001) analyzed the collagen weave of OPN^+/+^ and OPN^−/−^ mice with induced MI, and similarly found disrupted collagen patterns in the myocardium of OPN^−/−^ mice. In post-MI OPN^+/+^ mice, large collagen fibers and thin collagen weave were increased in non-infarct LV regions. However, post-MI OPN^−/−^ mice revealed a reduction in both of these types of collagen; Northern analysis demonstrated no significant change in collagen I (α1) mRNA expression. These data, combined with similar results using other models of myocardial remodeling (i.e., aldosterone infusion, streptozotocin diabetic cardiomyopathy, Ang II infusion) suggest that increased OPN expression in the heart post-injury plays a crucial role in regulating LV remodeling, at least in part, by supporting collagen synthesis and ECM functionality.

Our results (Fig. 4) indicate that ROS-induced OPN expression contributes to cardiac interstitial collagen accumulation in vivo. This response is well documented in the literature (Klingel and Kandolf, 2010; Zheng and Blobel, 2010); here we present evidence that APAP negatively regulates OPN mRNA levels in response to DOX in myocytes (Fig. 3). Together these results suggest that APAP inhibits increased fibrosis through negative regulation of collagen synthesis and extracellular matrix deposition.

### GATA4

**Fig. 6:**
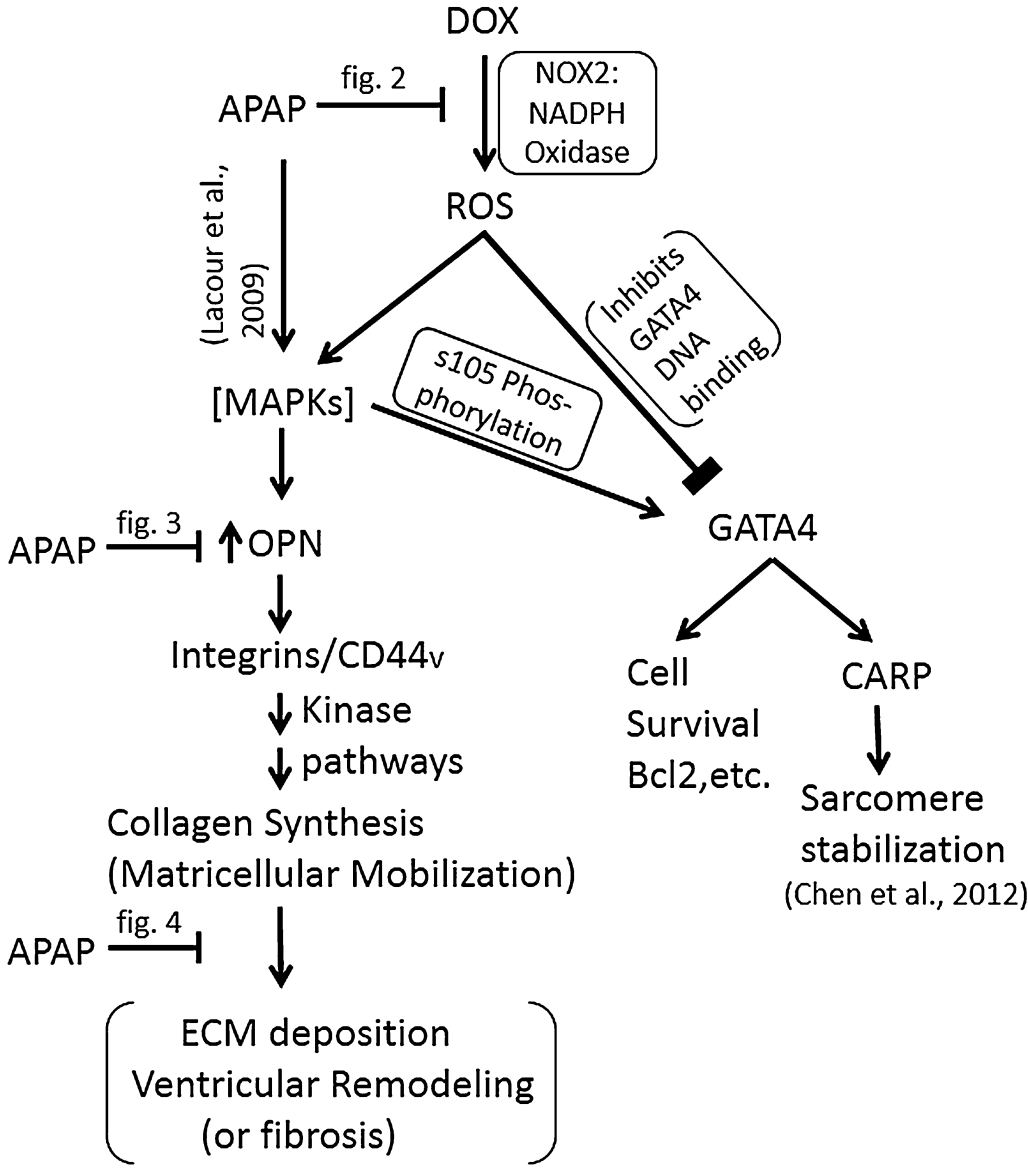
Anti-inflammatory action of APAP on DOX-induced cardiac remodeling. APAP diminishes DOXinduced ROS formation, thereby directly inhibiting down-stream signaling that leads to ventricular remodeling and fibrosis. It also preserves GATA4 activity, cell survival and sarcomere structure. Individual steps in the pathways and details of the two references are discussed in the GATA4 section of the text. The net result of APAP action is to reduce the harmful actions, notably fibrosis, of DOX caused by increased ROS levels.

GATA4 is a member of the zinc finger transcription factor family, important in regulating differentiation, sarcomere synthesis, and cell survival; it is expressed in the heart and regulates several cardiac-specific genes and a number of anti-apoptotic genes (Charron and Nemer, 1999; Charron et al., 1999; Aries et al., 2004). GATA4 is also a survival factor for differentiated postnatal cardiac myocytes (Kobayashi et al., 2009) and an upstream activator of the anti-apoptotic gene Bcl2. Many GATA4-regulated genes, including cardiac adriamycin-responsive protein (CARP), atrial natriuretic peptide, troponin I, and alpha-myosin heavy chain, are suppressed by anthracycline treatment, suggesting an interaction between DOX and GATA4 activity (Chen et al., 2007; Murphy et al., 1997). The cartoon in Figure 6 illustrates some of the complexity of the DOX-induced cascade of events. DOX-induced ROS down-regulates GATA4 DNA-binding activity in isolated rat cardiac myocytes and the HL-1 cardiac cell line, and ectopic expression of GATA4 attenuates the incidence of apoptosis (Kim et al., 2003). GATA4 contains a conserved MAPK phosphorylation site at serine 105 within the transcriptional activation domain; it is phosphorylated in response to agonist stimulation through ERK1/2, and weakly through JNK or p38 MAPKs (Liang et al., 2001). We showed (Fig. 5) that phosphorylation at serine 105 of GATA4 is decreased in response to DOX in OPN^+/+^ but to a lesser extent in OPN^−/−^ mice. This action is significantly attenuated in the presence of APAP, which can activate MAPKs (Lacour et al., 2009), thus maintaining the activity of GATA4. Sarcomere stabilization is mediated via CARP, a transcriptional regulatory protein that together with GATA4 regulates sarcomere gene expression (Chen et al., 2012).

Keeping this complex cell physiological process functioning smoothly is apparently quite a challenge considering that heart failure is a frequent cause of death. As a consequence of our studies and those of a number of other groups (e.g., Zhu et al., 2006) suggesting that acetaminophen has cyto-protective and anti-inflammatory actions in certain situations, the two senior authors of this paper routinely take Tylenol to reduce inflammation and protect tissues.
